# Neuroimmune Dynamics in Diseases of the Arterial Wall: Mechanistic Foundations and Translational Prospects

**DOI:** 10.1007/s11883-026-01418-y

**Published:** 2026-05-07

**Authors:** Qi Guo, Peter Holicek, Laura Tarnawski, Peder S. Olofsson

**Affiliations:** Laboratory of Immunobiology, Division of Cardiovascular Medicine, Department of Medicine, Karolinska Institutet, Solna, Stockholm, Sweden

**Keywords:** Aneurysm, Innervation, Neurovascular crosstalk, Vascular smooth muscle cell, Macrophage, Inflammation neuroscience

## Abstract

**Purpose of Review:**

Peripheral nerves are increasingly recognized as regulators of vascular pathology, shaping arterial tone, inflammation, and structural remodeling. This review delineates how neural circuits interface with the aortic wall, considers sympathetic and sensory pathways in atherosclerosis and aneurysm pathophysiology, and discusses emerging experimental approaches and therapeutic strategies.

**Recent Findings:**

Autonomic and sensory neural circuits are increasingly recognized as modulators of arterial disease. In experimental atherosclerosis, neuroimmune crosstalk shapes vascular inflammation and influences plaque stability. Limited available evidence from human and experimental studies suggest increased sympathetic innervation within aneurysm tissue. In preclinical models, aneurysm remodeling was linked with sympathetic input, and interventions that reduce noradrenergic signaling via sympathetic denervation or pharmacological adrenergic blockade attenuated disease severity. Improved understanding of the role for innervation in vascular pathophysiology may open therapeutic opportunities, including neuromodulation and pharmacological interventions. Clarifying sources of heterogeneity between models and clinical data can potentially refine therapeutic targets and patient selection, and advance opportunities for precision interventions.

**Summary:**

Peripheral neural circuits are integral to vascular homeostasis in health and disease, interfacing with blood vessels and regulating their physiology. Converging human and preclinical evidence implicates sympathetic innervation in disease development, and that dampening adrenergic signaling via denervation or adrenergic blockade may mitigate disease progression. This review discusses mechanistic neuroimmune crosstalk across atherosclerosis and aneurysm biology and outlines some potential translational opportunities. Together, the advances position neurovascular crosstalk as a potentially tractable axis for disease-modifying interventions.

## Introduction

**Fig. 1:**
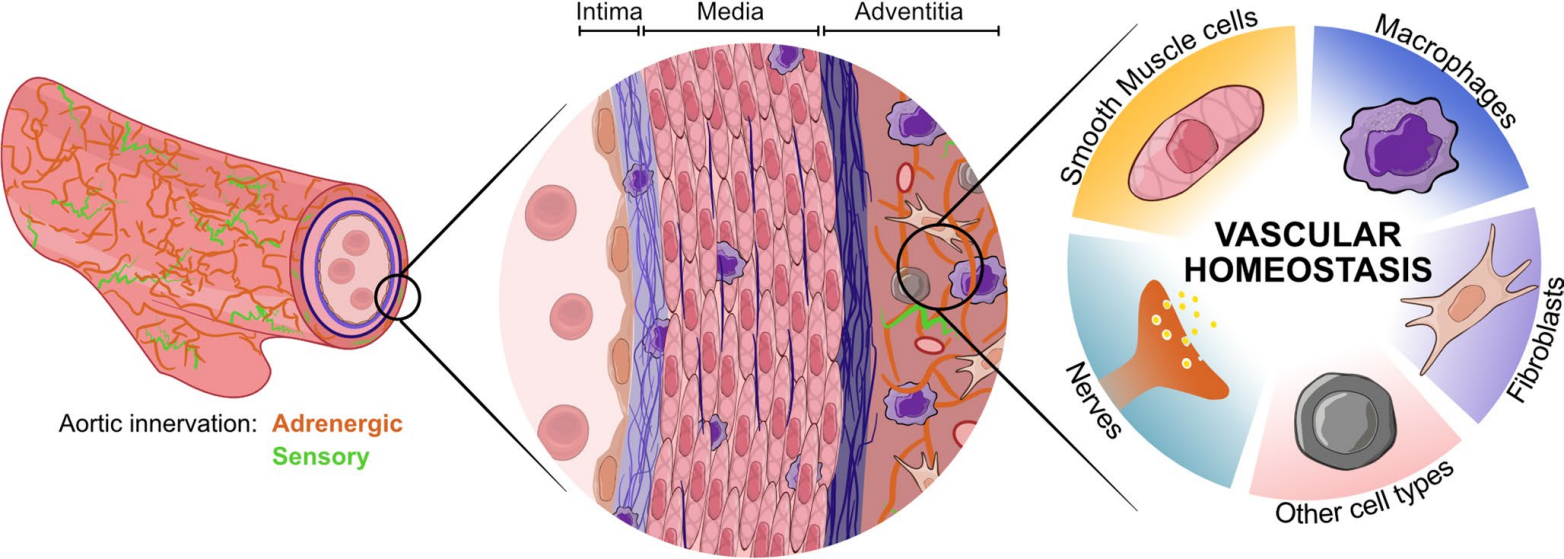
Conceptual model of neuro-vascular–immune crosstalk: Coordinated interactions among perivascular nerves, smooth muscle cells, fibroblasts, endothelial cells, and immune cells maintain vessel wall homeostasis; disruptions and maladaptation may contribute to pathological remodeling. (Original figure)

Vascular tissue homeostasis and adaptation rely on dynamic crosstalk among vascular cells, immune cells, and vascular innervation [1–8] (Fig. 1). These interactions influence multiple aspects of vascular pathophysiology, as evidenced by observations in atherosclerosis [1], autoimmune vasculitis [9] and aneurysm formation [10–12]. However, the mechanistic roles of these intercellular networks, particularly how aortic innervation contributes to vascular homeostasis and disease, remain poorly understood. This review discusses recent evidence on the neuro-immuno-vascular interactions and their contribution to vascular pathobiology.

The functional demands on the aortic wall require specialized architecture, including structural and spatial heterogeneity. The structural integrity and function of the aortic wall is sustained by a combination of structural layers (tunica intima, tunica media, and tunica adventitia), cellular components, and the extracellular matrix [13]. The regional heterogeneity across the aorta also arises from distinct developmental origins [13–17] and biomechanical environments [18]. Aortic innervation mirrors this heterogeneity [19]. Preganglionic sympathetic neurons (T1-L2) synapse in paravertebral and prevertebral ganglia, with the postganglionic fibers reaching the abdominal aorta via celiac, aorticorenal, and hypogastric plexuses. These fibers form periarterial networks in the adventitia [20, 21], adjacent to vasa vasorum, fibroblasts, and resident immune cells [1, 22]. Sympathetic terminals have the capacity to release norepinephrine (NE) and dopamine, and cognate receptors are expressed by cells in the adventitia, media and intima [23, 24]. Sensory afferents from thoracic and upper lumbar dorsal root ganglia accompany the autonomic fibers and can be found in the aortic adventitia [1]. In response to local environmental cues, sensory fibers have the capacity to release neuropeptides with potent vasoactive and immunomodulatory effects, such as calcitonin gene‑related peptide (CGRP) and substance P [25–31]. Cholinergic innervation of the aorta has also been reported [32] although its significance beyond the well-known functions related to the carotid body and aortic arch remains unclear. Interestingly, there is evidence that cholinergic signals are supplied by acetylcholine-producing lymphocytes expressing choline acetyltransferase (ChAT) [33, 34], where ChAT^+^ T cells can promote vasodilation through actions on vascular endothelial cells [35–37].

An important aspect of the adventitial innervation is the likely interactions between the peripheral nervous system (PNS) and resident and infiltrating immune cells carrying receptors for neuropeptides and neurotransmitters [38]. Distinct macrophage populations with context-dependent phenotypes have been associated with peripheral nerves in multiple tissues [39]. Experimental data supports reciprocal neuron-associated macrophage (NAM)-nerve crosstalk which shapes function on both sides and maintains homeostasis across multiple tissues [22, 40]. In adipose tissue, NAMs limit NE availability for thermogenic signaling and promote weight gain [41]. Similarly, a subset of NAMs is located close to sympathetic nerve fibers in the airways of the lungs and can regulate inflammation during viral infections [42]. In the kidney, a subset of macrophages associated with sympathetic nerves regulate salt and water homeostasis through control of local NE levels and renal sympathetic activity [43]. The aorta harbors tissue-resident macrophages across its layers, but the understanding of the potential neuroimmune crosstalk in the vasculature is limited at present [44, 45].

## Aortic Innervation in Vascular Pathophysiology

### Atherosclerosis

Breakthroughs in vascular pathophysiology have emerged from combining insights across disciplines to unravel complex disease mechanisms. For example, combining metabolism and immunology transformed our mechanistic understanding of atherosclerosis, revealing that systemic and local inflammation contribute to its pathophysiology and outcomes [44, 46–48]. The discoveries on inflammation have enabled new avenues for development of additional therapeutic approaches [49]. Already decades ago, observations linking heart rate variability, often considered to be an indirect marker of vagal nerve activity, and cardiovascular risk [50–52] sparked interest in the potential contribution of neural reflex activity in the pathophysiology of atherosclerosis. The eventual incorporation of neuroscience into atherosclerosis research uncovered roles for arterial innervation in vascular inflammation [53] and neural reflexes in plaque development [1, 54]. Evidence from experimental models indicates that attenuating adrenergic innervation can lessen plaque burden and favor a more stable phenotype, supporting a functional contribution of neurovascular crosstalk in the pathophysiology of atherosclerosis.

Innervation by Cgrp^+^ and Trpa1^+^ sensory neurons of the rodent aorta has been described [55, 56]. Although their functional significance remains incompletely understood, CGRP can exert vaso-regulatory effects via vascular smooth muscle cells and endothelial cells [27, 29, 57] and Trpa1 has been implicated in hemodynamic regulation [58]. CGRP can have context‑dependent pro‑ and anti‑inflammatory activities, including promotion of pro‑inflammatory cytokine production and mast‑cell degranulation, as well as inhibition of leukocyte adhesion and downregulation of pro‑inflammatory cytokine production [30]. Consistent with these observations, genetic deletion of α-CGRP in atherosclerosis-prone ApoE deficient mice aggravated atherosclerotic plaque development [31]. These observations nominate CGRP and Trpa1 signaling as modulators of atherogenesis and potential targets for further mechanistic exploration in the pathophysiology of atherosclerosis.

In addition to the observations that direct innervation controls important aspects of the vascular pathophysiology in atherosclerosis, there is evidence that other neural signals and neurotransmitters play a role in plaque development. As an example, genetic ablation of the α7 nicotinic acetylcholine receptor subunit (α7nAChR) in hematopoietic cells significantly increased experimental atherosclerosis while treatment with a α7nAChR ligand reduced it [59, 60], highlighting cholinergic signaling in modulation of vascular inflammation and atherogenesis, either through systemic or local mechanisms. This work is inspiring further exploration of neuro-immuno-vascular interactions.

### Aneurysm

Aortic aneurysms are characterized by a progressive, localized dilation of the aorta, typically asymptomatic, which can ultimately lead to life-threatening rupture. The pathophysiology of abdominal aortic aneurysm (AAA) is multifactorial and involves local inflammation, immune cell infiltration, oxidative stress, phenotypic modulation and apoptosis of vascular smooth muscle cells (VSMC), neovascularization, and extracellular matrix remodeling. This extracellular matrix degradation is in part mediated by matrix metalloproteinases (MMPs) [61, 62] as well as elastolytic cysteine proteases [63, 64], largely released from infiltrating immune cells. The pathogenesis of AAA has been extensively reviewed elsewhere [65, 66]. Therapies include anti-hypertensives, such as β-blockers, but their efficacy has not been unequivocally determined [67]. Definitive treatment is surgical or endovascular repair.

A recent study found increased expression of tyrosine hydroxylase (TH) and dopamine β-hydroxylase in biopsies from aortic aneurysms from both patients and experimental animals, suggesting enhanced sympathetic signaling within the diseased aortic wall [11]. The authors also reported that perivascular sympathetic remodeling in AAA promoted vascular smooth muscle cell phenotypic switching toward a synthetic and more pro-inflammatory state. The process involved osteoclast-like macrophage-derived Semaphorin 4D, promoting sympathetic nerve sprouting and ATP-mediated neurovascular signaling, accompanied by aneurysm progression. In the same study, ablation of sympathetic TH+ nerves by systemic 6-hydroxydopamine injection or celiac ganglionectomy attenuated aneurysm growth [11]. Another experimental study of angiotensin II-induced abdominal aortic aneurysms showed increased sympathetic axon density, increased levels of norepinephrine and aortic stiffness, and co-localization of axons and macrophages in the aortic adventitia as compared with sham-treated mice. The effects were attenuated in mice subjected to voluntary wheel running, indicating that exercise may mitigate some of the adverse effects on aortic physiology by angiotensin II in this model [68]. Supporting this concept, a phenol-based abdominal aortic denervation model demonstrated that local neural ablation is sufficient to induce pronounced aortic remodeling, including reduced expression of contractile VSMC markers, elastin fragmentation, and altered adventitial architecture. It should be noted that phenol induces non-selective neural ablation and may have additional local effects. While a significant reduction in TH+ nerve fibers was confirmed, parallel disruption of other perivascular components is likely in this model [69]. Together, the reported effects of interfering with sympathetic signaling in the context of aortic aneurysms raise intriguing questions about whether targeting neurovascular interactions can influence aneurysm growth, a hypothesis that merits further exploration.

The experimental and limited clinical evidence on AAA pathophysiology supports that sympathetic innervation is an important determinant of aneurysm pathophysiology, but the influence of other neural components has not been systematically mapped. Sensory nerves innervate the vasculature [56] as discussed previously, but little is known about their presence and potential pathophysiological importance in the context of aortic aneurysm formation. For example, whether aneurysm development involves remodeling of the artery-associated sensory innervation and neuropeptide expression is unknown. Further, the role of a number of potent vasoactive mediators, including vasoactive intestinal peptide (VIP), modulate smooth muscle relaxation and immune effects in other organs, is yet incompletely understood in aneurysm biology [70].

Together, these observations highlight the complex interplay between innervation, vascular smooth muscle cell plasticity, and immune cell dynamics in the pathogenesis of aortic aneurysms. Future studies should delineate the mechanistic roles of innervation, not least the sensory, and nerve-associated macrophages in aneurysm pathobiology, as this knowledge could open novel strategies to explore for disease prevention and intervention.

## Neuroimmune Modulation in Vascular Disease: Tentative Therapeutic Directions

Beyond surgical or endovascular correction, therapeutic approaches that limit the progressive growth of aortic aneurysm and reduce the risk of rupture with convincing efficacy are currently largely lacking [66]. Genetic and observational studies have suggested that treatment with e.g. interleukin-6 antagonists, agents that reduce low-density lipoprotein cholesterol, metformin, or ACE-inhibitors are associated with improved outcomes, but the observations remain to be prospectively evaluated in randomized trials. Treatment with antibiotics, anti-hypertensives, fibrates and other drugs have been evaluated in clinical trials, yet without consistently convincing positive effects [66]. Neurovascular remodeling, including sympathetic sprouting and adrenergic signaling, appears to be associated with elevated pro-inflammatory factors and synthetic phenotypes in VSMCs [11]. Moreover, macrophage populations apposed to peripheral nerves may modulate cytokine gradients and neurotransmitter availability, thereby potentially reshaping the local immune and neurochemical microenvironments in multiple tissues [22, 40]. Collectively, the observations suggest that neuroimmune crosstalk is an integral part of both the pathophysiology of atherosclerosis and aortic aneurysm progression, at least in experimental settings. Accordingly, targeting the neurovascular–immune interface merits cautious consideration as part of future therapy development for these vascular conditions.

One therapeutic approach that in other contexts than cardiovascular inflammation has been used to reduce systemic levels of pro-inflammatory cytokines such as TNF and IL-1, desirable in treatment of prevalent cardiovascular diseases [49, 71], is intermittent electrical activation of the left cervical vagus nerve stimulation (VNS) using implanted devices. VNS is interesting because electrical activation of the cervical vagus nerve reduces systemic levels of pro-inflammatory cytokines and inflammation across a wide range of experimental animal models [4, 72–79] as well as in a number of small clinical studies of inflammatory diseases [76, 80–83]. The therapeutic effect of VNS has some similarities with administration of anti-cytokine drugs. One notable example is that VNS significantly reduced clinical disease scores in a recent randomized and placebo-controlled multi-center study of patients diagnosed with rheumatoid arthritis and insufficient therapeutic response to established therapy [84]. Interfacing with the vagus nerve in this way appears to be safe and feasible: A large number of patients with difficult-to-treat epilepsy were implanted with a stimulator for electrical activation of the cervical vagus nerve [85]. To date, the main reported adverse events include surgical-wound infections and temporary voice hoarseness due to activation of the recurrent laryngeal nerve. Whether reducing systemic levels of pro-inflammatory cytokines in cardiovascular diseases of inflammatory origin using VNS may be beneficial for clinical outcomes or not remains to be evaluated.

Despite the observed efficacy of implanted vagus nerve stimulators for reduction of inflammation, results with non-invasive methods that attempt electrical nerve activation using skin electrodes appear less convincing in patient studies [86]. Notably, reports of noninvasive stimulation often lack confirmation of target nerve engagement. There is therefore an urgent need to evaluate non-invasive approaches in terms of their ability to activate the target nerves in clinical contexts before such devices can be reliably used in clinical studies in the contexts of inflammatory and cardiovascular diseases.

In the context of arterial aneurysms, it has been reported that transcutaneous electrical stimulation above a sensory branch of the vagus nerve in the right outer ear reduced the risk for intracranial aneurysm rupture and reduced mRNA expression of MMP-9 in a network of arteries at the base of the brain in experimental mice [87], but the mechanism behind these observations remains unknown. Nonetheless, the findings that application of an electric field using electrodes on the surface of the skin has effects on arterial pathophysiology is intriguing, and warrants further validation and mechanistic evaluation.

If increased cholinergic activity is desired, administration of inhibitors of acetylcholine esterase may be an option. In patients diagnosed with metabolic syndrome, short-term treatment with the acetylcholine esterase-inhibitor galantamine reduced inflammation and insulin resistance in a randomized trial [88], supporting the hypothesis that cholinergic signals can reduce aspects of cardiovascular inflammation and highlighting the potential utility of pharmacological approaches also in this context.

As our understanding of the mechanisms and pathophysiological roles of vascular neuroimmune interactions deepens, exploring therapeutic strategies that combine systemic pharmacological approaches with selective modulation of neural signals, leveraging their respective strengths in systemic and anatomically targeted effects, may prove valuable. Improved non-invasive methods for nerve activation are being developed with the aim of achieving better precision targeting. Some approaches use combinations of multiple electrical fields, ultrasound, or other innovative technologies [89–91]. Achieving non-invasive precision targeting of the nervous system holds promise to advance novel aspects of clinical cardiovascular research and potentially accelerate the translation of the expanding insights from experimental studies into how neuroimmune interactions influence vascular function and contribute to cardiovascular pathophysiology.

Rapidly evolving technological innovations promise to enhance our ability to study visceral functions, particularly in experimental settings [92–95]. There is hope that new technologies will unlock novel avenues for interventions and enable local vascular neuromodulation and ultimately closed‑loop regulation of vascular neuroimmune crosstalk for improvement of treatment outcomes. However, we are most likely yet only scratching the surface on the pathophysiological effects of neuroimmune interactions in cardiovascular diseases. Methods for targeting cholinergic, sympathetic, and sensory signals with limited precision are already available both using pharmacological and device-based approaches [93, 94] but their lack of specificity is a significant challenge not least for mechanistic interpretation of outcomes. Ongoing refinement of experimental methods promises to enable addressing of pressing scientific questions, including functional mapping of vascular innervation and homeostatic reflexes in health and disease, and their involvement in neural regulation of inflammation, metabolism, and development. Going forward, it will be important to define assayable signatures of inadequate neuroimmune crosstalk and to develop interventions that can achieve verifiable target engagement with predictable therapeutic effects. A better understanding of neural regulation of cardiovascular pathophysiology may add an additional layer of mechanistic insights into disease mechanisms that could improve both diagnostic and therapeutic options. Accordingly, it appears important to further deconvolute the involvement of neuro-immune crosstalk in cardiovascular diseases.

## Conclusion

The arterial adventitia is a dynamic neuro-immune hub, functionally integrated with vascular physiology and influencing immune- and smooth muscle cell function, extracellular matrix integrity, and other key processes in atherosclerosis and aneurysm biology. Changes is the vascular innervation are linked to disease development and disruptions of homeostasis, highlighting the need to improve our mechanistic understanding of vascular neuro-immune crosstalk and its potential effects in cardiovascular pathology. Advances in imaging, genetics, and bioelectronic technologies now provide opportunities to functionally map h neuro-immune interactions in the vascular microenvironment as well as connections to neural reflex arcs, instights that may potentially support development of more precise, targeted interventions. Translational success will be facilitated by improving spatial, temporal, and functional targeting of these neuroimmune interfaces and disease-modifying signaling pathways. Thus, improving our understanding of neural regulation in cardiovascular disease may yield additional mechanistic insight to inform refinement of diagnostic and therapeutic strategies, highlighting the need for continued efforts to delineate neuro-immune crosstalk.

## Key References

- Mohanta SK, Peng L, Li Y, Lu S, Sun T, Carnevale L, et al. Neuroimmune cardiovascular interfaces control atherosclerosis. Nature. 2022;605:152–159. https://doi.org/10.1038/s41586-022-04673-6
  - ◦ This study demonstrated neural control of plaque composition and development in experimental atherosclerosis, and described the atherosclerosis-brain circuit.
- Guimaraes EL, Dias DO, Hau WF, Julien A, Holl D, Garcia‑Collado M, et al. Corpora cavernosa fibroblasts mediate penile erection. **Science**. 2024;383:eade8064. https://doi.org/10.1126/science.ade8064
  - ◦ This study links neural activity and neurotransmitter release with arterial function.
- Tang Z, Xie J, Jin M, Wei G, Fu Z, Luo X, et al. Sympathetic hyperinnervation drives abdominal aortic aneurysm development by promoting vascular smooth muscle cell phenotypic switching. J Adv Res. 2025;71:383–398. https://doi.org/10.1016/j.jare.2024.05.028
  - ◦ This study linked sympathetic innervation to structural degeneration in an animal model of abdominal aortic aneurysms.
- Cho JM, Vu K, Park SK, Zhu E, Zhao P, Yokota T, et al. Habitual exercise modulates neuroimmune interaction to mitigate aortic stiffness. Circ Res. 2025;136:1579–1594. https://doi.org/10.1161/circresaha.124.325656
  - ◦ This study revealed that long-term habitual exercise reshapes neuroimmune interactions within the aortic wall, reduced vascular inflammation and attenuated aortic stiffness in experimental animals, linking neuroimmune regulation to vascular remodeling.
- Zhu Q, Xiao L, Cheng G, He J, Yin C, Wang L, et al. Self-maintaining macrophages within the kidney contribute to salt and water balance by modulating sympathetic nerve activity. Kidney Int. 2023;104:324–333. https://doi.org/10.1016/j.kint.2023.04.023
  - ◦ This study showed that macrophages  regulate neural activity that control homeostasis.
- Golledge J, Thanigaimani S, Powell JT, Tsao PS. Pathogenesis and management of abdominal aortic aneurysm. Eur Heart J. 2023;44:2682–2697. https://doi.org/10.1093/eurheartj/ehad386
  - ◦ A comprehensive review of abdominal aortic aneurysm pathogenesis and clinical management.

## Data Availability

No datasets were generated or analysed during the current study.
